# Non-Alcoholic Fatty Liver Disease and Nutritional Implications: Special Focus on Copper

**DOI:** 10.3390/nu9101137

**Published:** 2017-10-18

**Authors:** Laura Antonucci, Cristiana Porcu, Gino Iannucci, Clara Balsano, Barbara Barbaro

**Affiliations:** 1 Francesco Balsano Foundation, 00185 Rome, Italy; antonuccilaura1986@libero.it (L.A.); cristiana.porcu@live.it (C.P.); clara.balsano@cc.univaq.it (C.B.); 2 Dipartimento di Medicina Interna e Specialità Mediche, Sapienza Università di Roma, 00185 Rome, Italy; gino.iannucci@uniroma1.it

**Keywords:** NAFLD, copper, ROS, nutrients, antioxidants, lipids, liver

## Abstract

Non-alcoholic fatty liver disease (NAFLD) is characterized by excess lipids in hepatocytes, due to excessive fatty acid influx from adipose tissue, de novo hepatic lipogenesis, in addition to excessive dietary fat and carbohydrate intake. Chronic hepatic lipid overload induces mitochondrial oxidative stress and cellular damage leading the development of NAFLD into a more severe liver disease condition, non-alcoholic steato-hepatitis (NASH). In turn, this can progress to cirrhosis and hepatocellular carcinoma (HCC). Among others, copper is one of the main bio-metals required for the preponderance of the enzymes involved in physiological redox reactions, which primarily occurs during mitochondrial respiration. Thus, copper homeostasis could be considered a target point for counteracting the progression of NAFLD. Accordingly, many diseases are correlated to unbalanced copper levels and, actually, some clinical trials are examining the use of copper chelating agents. Currently, no pharmacological interventions are approved for NAFLD, but nutritional and lifestyle modifications are always recommended. Fittingly, antioxidant food agents recognized to improve NAFLD and its complications have been described in the literature to bind copper. Therefore, this review describes the role of nutrition in the development and progression of NAFLD with a particular focus on copper and copper-binding antioxidant compounds against NAFLD.

## 1. Introduction

Non-alcoholic fatty liver disease (NAFLD) consists of fat accumulation in the liver (hepatic steatosis) and affects about 1.8 billion people [1]. NAFLD can progress towards non-alcoholic steato-hepatitis (NASH), which is a more severe condition characterized by necro-inflammation with or without fibrosis. This can ultimately lead to cirrhosis and hepatocellular carcinoma (HCC). In addition, the related cardiovascular risk cannot be underestimated [2]. To note, NAFLD is considered the hepatic manifestation of the metabolic syndrome, which is mainly characterized by obesity, dyslipidemia, insulin resistance, hypertension, and type 2 diabetes. In particular, in asymptomatic morbidly obese patients, there is a very high prevalence of approximately 90% of NAFLD. Of these morbidly obese patients with NAFLD, more than one-third fit the histological criteria for NASH [3,4]. Taken together, all these conditions have resulted in NAFLD being considered an important problem for the health burden nationwide.

The excess of lipids in hepatic steatosis primarily constitutes triglycerides (TGs) and cholesterol esters, which are stored in dynamic organelles called lipid droplets (LDs) [5].

Steatosis has been pathologically classified as macrovesicular steatosis, characterized by the formation into the hepatocytes of large droplets which displaces the nucleus, and microvesicular steatosis, the accumulation of small fat droplets with preserved cellular architecture, but corresponding to a more severe condition significantly associated to cellular injury. In particular, microvesicular steatosis displays “megamitochondria” with severe impairment of the mitochondrial β-oxidation, which is the main catabolic process by which fatty acids are broken down to produce energy [6]. The consequently increased generation of reactive oxygen species (ROS) induces lipid peroxidation, which leads to liver inflammation and fibrogenesis. Therefore, oxidative stress plays a major role in the progression of NAFLD, which seems to be the most important mechanism leading to hepatic injury. Bio-metals play a key role in controlling ROS formation and amongst the others, copper is one of the most important bio-metals, especially for the enzymes involved in mitochondrial respiration.

Hence, the aim of this review is to discuss the pathogenesis and worsening of NAFLD in relation to nutrition, with a special regard to copper and to copper-binding natural antioxidant compounds against NAFLD.

## 2. Pathophysiology of NAFLD and Nutritional Implications

The pathophysiology of NAFLD is complex as many factors contribute to fat deposition within the liver. These include endogenous factors, such as excessive fatty acid influx from fat depots (mainly white adipose tissue) or de novo hepatic lipogenesis from non-lipid precursors, and exogenous factors, such as excessive dietary fat and carbohydrate intake. Thus, beyond the metabolic intrinsic disorders, it is of critical importance to understand how diet and nutrient composition impacts on the development of NAFLD. Western diet contributes to NAFLD pathogenesis since it is excessively rich in fats and carbohydrates. Here, we will describe the main features of dietary nutrients involved in the onset of hepatic steatosis.

### 2.1. Dietary Fats

Dietary fats are essentially TGs, which are hydrolyzed by lipases to diacylglycerols (DG), monoacylglycerols (MG), and fatty acids (FAs) in the intestinal lumen, and taken up by the enterocytes. In the enterocytes, the FAs are again packaged as TGs or cholesterol esters in chylomicrons for the delivery through the bloodstream to peripheral tissues. Finally, the remaining chylomicron remnants are delivered to the liver. Their uptake into hepatocytes is mediated by the low density lipoprotein (LDL) receptor and LDL receptor-related protein (LRP) [5]. In the hepatocytes, FAs can be esterified or oxidized, and accumulated or secreted [7].

FAs can be present as saturated and mono- (MUFAs) or poly-unsaturated fatty acids (PUFAs): MUFAs are fatty acids that have one double bond in the fatty acid chain with the remaining carbon atoms being single-bonded, while PUFAs contain more than one double bond in their backbone. Their relative proportions determine different net biological effects.

Puri et al. [8] performed a lipidomic profile of total plasma and hepatic lipids of NAFLD and NASH patients showing that the concentration of saturated and MUFA were higher in individuals with fatty liver compared to healthy controls. Consistent with these results, Toshimitsu et al. [9] highlighted that patients with steatosis and NASH present a lower dietary ratio of PUFA/saturated fatty acids compared to that of healthy subjects. This association between the fatty acid ratio and the severity of fatty liver disease may be due to various molecular mechanisms, among which oxidative stress plays a predominant role. Specifically, a correlation between the intake of saturated fatty acids and impaired glutathione metabolism was found, suggesting the deleterious pro-oxidant effects of saturated fatty acids [10]. Accordingly, it has been shown that a diet enriched in *n-*3 PUFA reduces the accumulation of hepatic TGs, restores insulin sensitivity, and ameliorates liver steatosis and the level of hepatic biomarkers (ALT, AST, and GGT) [11,12]. Specifically, *n-*3 PUFA, which are the so-called omega-3 fatty acids mainly present in fish oil, are the natural ligands of peroxisome proliferator-activated receptor α (PPARα), which is a master regulator that promotes fatty acid β-oxidation in mitochondria. Mitochondrial β oxidation not only provides energy for hepatocytes, but also generates ketone bodies providing metabolic fuels for extrahepatic tissues during fasting. Low levels of circulating *n-*3 PUFA with a consequent increase of the *n-*6/*n-*3 FA ratio impairs PPARα activity in the liver. This phenomenon is associated with a higher hepatic uptake of circulating FAs, a decrease in mitochondrial lipid β-oxidation, and an up-regulation of lipogenic transcription factors, as well as, first, the sterol regulatory element binding protein-1 (SREBP-1) [13].

### 2.2. Dietary Carbohydrates

During the fed state, the liver has a crucial role in storing excess carbohydrates as lipids via de novo lipogenesis. A high-carbohydrate diet can prime de novo lipogenesis since the abundance of acetyl-coenzyme A (acetyl-CoA) derived from glycolysis can be used as the substrate for synthesizing long-chain FAs. High glucose intake and the consequent insulin secretion regulates several factors, such as carbohydrate response element binding protein (ChREBP) and SREBP1, enables the expression of lipogenic genes, such as fatty acid synthase (FASn). FAs derived from de novo lipogenesis are esterified by a series of enzymatic reactions, culminating in formation of TGs that will be stored in lipid droplets [14]. In the case of hyperinsulinemia, insulin continues to drive lipogenesis via the SREBP1 pathway in addition to failing to suppress gluconeogenesis, contributing to exacerbating hepatic steatosis [15].

During the last decade, dietary habits have evolved to favor unhealthy high fatty food and sweetened foods, such as many beverages. It is known that the increased intake of carbohydrates increases the risk for fatty liver, metabolic syndrome, type 2 diabetes, obesity, and cardiovascular diseases, which is likely due to an excessive caloric intake [16]. In this regard, it is important to underline that the presence of a high quantity of fructose together with the alteration in the ratio between saturated and unsaturated fatty acids is crucial in the onset and progression of NAFLD, which is associated with increased oxidative stress and insulin resistance. Many experimental studies on animal models and epidemiological studies in human subjects linked the excessive consumption of fructose to NAFLD to its progression and severity of fibrosis [17]. Dissimilar to glucose, hepatic metabolism of dietary fructose is independent of energy status which results in unregulated hepatic fructose uptake that finally leads to an uncontrolled increase of lipogenesis [14]. Specifically, fructose stimulates PPARγ-coactivator-1β (PGC-1β), which acts as a co-activator of SREBP1c. Moreover, fructose inhibits hepatic FA β-oxidation, which mainly occurs by inhibiting the transcriptional activities of PPARα. Thus, the shift towards lipogenesis over FA oxidation contribute to hepatic steatosis and, hence, in a vicious circle, to insulin resistance [18,19].

## 3. NAFLD-Related Oxidative Stress

Chronic lipid and carbohydrate overload in the liver in individuals with NAFLD induces mitochondrial oxidation, which results in oxidative stress and eventual damage to cellular components, including mitochondria. This can cause cell death and inflammation, which signals the progression from benign steatosis to NASH [20]. Sunny et al. found a positive correlation between intrahepatic TGs levels and mitochondrial oxidative metabolism, which was approximately two-fold greater in NAFLD patients [21]. The constitutive over-activation of oxidative metabolism during NAFLD causes mitochondrial damage: the oxidative stress associated with elevated hydride production in the tricarboxylic acid (TCA) cycle may be sufficient to damage the electron transport chain with impaired adenosine triphosphate (ATP) synthesis. Accordingly, patients with NASH have decreased expression of mitochondrial DNA (mtDNA)-encoded polypeptides and low activity of complexes I, III, IV, and V [22]. The consequently increased generation of ROS and reactive aldehydic derivatives causes oxidative stress and cell death via ATP, nicotinamide adenine dinucleotide (NAD), and glutathione depletion, in addition to the consequent damage to DNA, lipids, and proteins. Furthermore, many studies on animal models have confirmed the effect of impaired mitochondrial activity on the onset of NAFLD and insulin resistance, which has prompted the scientific community to consider NAFLD as being mainly a mitochondrial disease [23,24,25].

### 3.1. Special Focus on Copper

Copper may be in an oxidized (CuII) or reduced (CuI) state, which allows it to accept or donate individual electrons. This property allows it to be a cofactor in many physiological redox reactions. Copper has essential roles in the mitochondrial electron transport chain (e.g., for cytochrome c oxidase); in the detoxification of ROS (e.g., for superoxide dismutase SOD); in neurotransmitter synthesis; as well as in the modulation of cellular energy metabolism and epigenetic modifications [26,27]. However, an excess of copper results in oxidative stress related health disorders: excess copper, in fact, it assists oxidative tissue injury through a free radical-mediated pathway. On the other hand, copper deficiency affects the antioxidant defense system resulting in increased ROS levels and the related oxidative damage of lipid, DNA, and proteins [28]. Thus, a highly-orchestrated regulation of copper pools is required to prevent oxidative stress and free radical damage events. Imbalances in physiological copper levels or tissue pathogenic compartmentalization, arising from genetic and/or dietary factors, are correlated with metabolic disorders, neurodegenerative diseases, and cancer [28,29].

#### 3.1.1. Copper Homeostasis and Metabolism

Mammals acquire copper from dietary sources, with drinking water and food typically high in copper, including meats, shellfish, seeds, beans, and cereals. Poor diet quality or malabsorption can have health consequences related to copper insufficiency [30].

**Figure 1:**
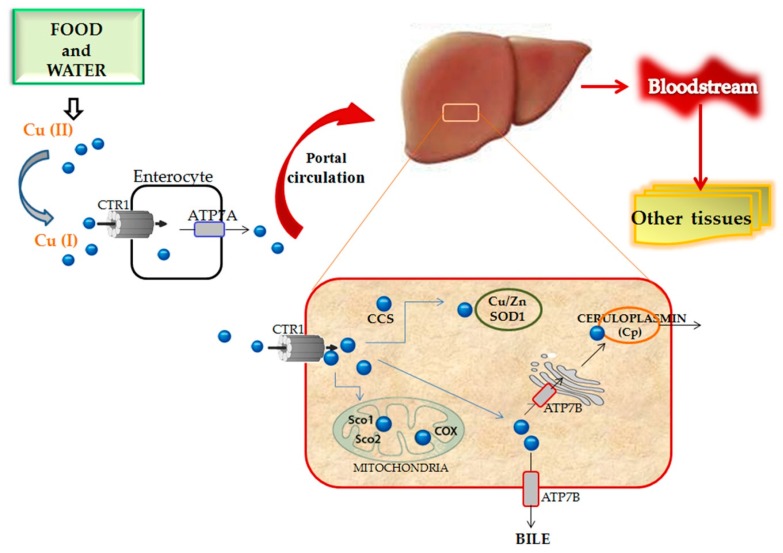
Copper absorption, distribution, and metabolism: copper enters the enterocytes through CTR1 and flows out in the portal circulation by ATP7A. In the liver, Cu has a key role in defense against ROS (binds SOD) and in mitochondrial respiration. Linked to CP, copper is brought in the bloodstream to be transported to other tissues and organs. ATP7B ensures copper transport across the membranes of cellular organelles or allows for excess copper to be excreted into the bile. Cu: copper; CTR1: copper transporter protein-1; CCS: copper chaperone of superoxide dismutase; COX: cytochrome-C oxidase; Sco1, Sco2: cytochrome c oxidase assembly factors; Cu/Zn SOD: copper-zinc-dependent superoxide dismutase; ATP7A/B: copper-transporting ATPase A/B.

Copper is absorbed mainly in the duodenum, although it is thought that some absorption takes place in the stomach and in the distal part of the small intestine. Copper uptake into enterocytes is mainly managed by the human copper transporter protein-1 (hCTR1). Dietary copper is in the Cu^2+^ form, but its absorption by hCTR1 occurs as Cu^+^. Thus, before its uptake, copper needs to be reduced by metallo-reductases. Studies using epitope-tagged CTR1 have shown that CTR1 undergoes constitutive recycling, with high copper exposure resulting in rapid endocytosis of this transporter [31,32,33]. The release of copper from the basolateral membrane of enterocytes to the bloodstream involves the copper-transporting ATPase ATP7A. Utilizing ATP, this pumps copper within the portal system to reach the liver, which represents the central regulatory organ of copper homeostasis [34]. In hepatocytes, copper is distributed to many different enzymes for many different purposes. For example, it is delivered to the radical-detoxifying enzyme copper-zinc-dependent superoxide dismutase (SOD1) by the copper chaperone of superoxide dismutase (CCS) to exert ROS detoxification; incorporated into cytochrome-c oxidase (COX) in mitochondria for mitochondrial respiration; integrated into the copper-dependent ferroxidase ceruloplasmin (CP), the major copper-carrying protein, to be secreted in the blood [35]. The latter is a process carried out by the Cu-ATPase ATPB, which is the corresponding enzyme of ATP7A located in the liver. Following this, copper can be taken up by other tissues, including brain, kidney, heart, connective tissue, and the pancreas [32]. Instead, the removal of excess copper from the liver is driven by the re-localization of ATP7B, which pumps copper into the bile [36]. ATP7A and ATP7B are also regulated by a continuous relocation. When copper levels are low, both ATP7A and ATP7B are situated in the trans-Golgi network (TGN) where they transport copper to copper-dependent enzymes. However, at elevated copper levels, both transporters re-localize to the plasma membrane to facilitate copper export [36] (Figure 1).

Copper homeostasis is known to be linked to iron and to zinc homeostasis. The dysregulation in one of these metals may lead to dysregulation of the others. The link between copper and iron is the role of copper in ferroxidases of ceruloplasmin and hephaestin, which oxidize iron for mobilization. This avoids the risk of radical generation in auto-oxidation [37]. The mechanistic interaction of copper and zinc is less well understood, but it is known that high zinc consumption inhibits copper absorption, as observed by acquired hypo-cupremia associated with excess zinc consumption [38]. Alterations in homeostasis of these metals often lower a cell ability to regulate redox conditions resulting in cellular damage [30].

#### 3.1.2. Copper and Its Role in NAFLD Onset and Progression

Evidence reports that inadequate copper intake may be involved in the pathogenesis of NAFLD [39]. Subjects with NAFLD present lower, or slightly lower, intrahepatic and serum copper concentrations with respect to other liver diseases. This is consistent with other literature, as dietary copper restriction in rats induces a modification in lipid metabolism with the development of hepatic steatosis and insulin resistance, which suggests that a low copper availability may be involved in the development of NAFLD [40]. Moreover, Tosco et al. performed a gene-microarray differential analysis on the intestinal transcriptome of copper- and iron-deficient rats, highlighting that copper deficiency downregulates the mitochondrial and peroxisomal beta-oxidation of FA [41]. Notably, systemic copper deficiency in mice causes mitochondrial dysfunction, which is indicative of a defective mitochondrial function. Similar morphological alterations have also been described in human NAFLD [25,35], highlighting the important link between copper, mitochondrial function, and NAFLD. Furthermore, it was observed in rats that a diet rich in fructose, but with a low copper level, triggers liver steatosis and damage. In fact, fructose also acts as an inhibitor of duodenal copper absorption and, thus, boosts the impairment of oxidant defense and lipid peroxidation [35].

The studies on copper levels in NASH patients were less uniform with respect to those on NAFLD. It was reported that low copper levels were also found in NASH patients, both in adults and in children [42]. Aigner et al. reported that NASH patients had lower intrahepatic copper concentrations with respect to NAFLD patients, but they did not find relevant differences in serum copper levels between the two groups [40]. Our recent data indicate that serum copper levels of NASH patients begin to rise from NAFLD to NASH and from cirrhosis to HCC [43]. Accordingly, Geetha et al. highlighted high levels of copper in serum and hepatic tissue of HCC patients correlated with high oxidative stress [35]. Furthermore, even if it is not clear, some emerging clinical reports indicate that Wilson’s disease, an autosomal recessive disease characterized by excess copper, is a risk factor for HCC, pointing out an oncogenic potential of excess copper [44,45]. Nevertheless, understanding a role of copper in the hepatic carcinogenesis requires more deep investigation. Furthermore, the mechanisms that lead to a change between low and high copper levels in NAFLD and HCC, respectively, need to be explored.

Nevertheless, this evidence implies that the deregulation of copper in NAFLD and the consequent oxidative stress, if not counteracted, could lead to serious damage.

#### 3.1.3. Copper and Nutrients in NAFLD

Good, and especially balanced, nutrition is the basis for good health. Choosing food more or less rich in copper can be useful during diseases characterized by altered copper homeostasis.

The recommended daily allowance (RDA) of copper for adults is 1–3 mg/day [46]. The soil Cu concentration has an influence on food products. The use of Cu compounds as bactericides or fungicides on crops and Cu emissions from smelting and casting industries may affect the Cu content in the harvest. Moreover, Cu concentration in drinking water has to be considered. Water is known to contain concentrations of a few micrograms to more than 2 mg/L, which may also vary depending on groundwater composition and household plumbing systems [47].

Copper is widely distributed in foods. The major contributors of dietary copper are, especially, meats, seafood, nuts, seeds, and cereals and whole grain products. Specifically, the top ten foods are beef liver, sunflower seeds, lentils, almonds, dried apricots, dark chocolate, blackstrap molasses, asparagus, mushrooms, and turnip greens [46].

Given that the oxidative stress has a key role in the pathogenesis of human NAFLD, it is likely that anti-oxidant molecules are an option for its treatment.

Antioxidant molecules contain mainly a polyphenolic structure and possess the ability to scavenge and react preferentially with ROS [48]. They can be found in a variety of commonly consumed products, which are mainly obtained from plant sources. Many anti-oxidant natural compounds are described to counteract NAFLD, its progression towards NASH and related complications [35,49,50,51].

Interestingly, many of those compounds are able to bind copper, highlighting a direct action on copper-related dysfunction. In this present review, we can only list the antioxidants described to act against NAFLD that have been described having copper-binding activity in separate studies. These include: curcumin, epigallocatechin-3-gallate (EGCG), Luteolin and Luteolin-7-Glucoside, Caffeic Acid and Caffeine, oleuropein, quercetin and rutin, resveratrol (3,5,4′-trihydroxy-*trans*-stilbene).

##### Curcumin

This is a polyphenol found mostly in rhizomes of *Curcuma longa*. It exhibits antioxidant and anti-inflammatory properties. Studies on animals showed that curcumin prevents dietary-induced hepatic steatosis and attenuates many of the pathophysiological processes involved in the development and progression of NASH. Furthermore, it counteracts the onset of fibrosis [52,53].

Its keto-enolic moiety enables this compound to bind copper and exerts its antioxidant and chelating activities [54,55].

##### Epigallocatechin-3-Gallate (EGCG)

This is a phenolic antioxidant found in a number of plants, mostly in green tea.

Studies on animals highlighted that EGCG was able to prevent obesity by stimulating the mitochondrial complex chain, thereby contributing to the prevention of hepatic steatosis and improved insulin sensitivity [56]. It was also described as a potent copper chelator [57].

##### Luteolin and Luteolin-7-Glucoside

These are flavones found in aromatic plants, such as oregano, parsley and salvia, but also in olives, artichokes, carrots, pomegranate, and cacao. Both Luteolin and Luteolin-7-Glucoside (L7G, the glycosylated form of Luteolin) are described as being useful in the control of NAFLD. They are able to decrease lipogenesis by downregulating SREBP1 and increase the lipid β-oxidation capacity of the liver by activating PPARα, which contributes to lower total and LDL cholesterol levels [58,59]. These molecules also share the property of binding copper [60].

##### Caffeic Acid and Caffeine

Many works have highlighted the beneficial effects of coffee, caffeine, and caffeic acid in preventing NAFLD [51]. Specifically, studies on animal models and epidemiological studies on humans reveal that coffee intake can reduce the risk of NAFLD [61]. A systemic review highlighted that regular coffee or caffeine consumption is significantly associated with reduced hepatic fibrosis in patients with NAFLD [62]. Moreover, in hepatic cells, caffeic acid was described to reduce lipogenesis and increase lipid β-oxidation, which leads to a reduced lipid accumulation [63]. Thesemolecules are described as being able to bind copper [64].

##### Oleuropein

This is found in olives and olive leaves, with recognized beneficial properties with respect to human health [65].

It has an important role in counteracting hepatic lipid accumulation [65] and the progression of NASH to fibrosis [66] in mice fed with a high-fat diet.

Notably, it is able to bind copper, which could intervene in its antioxidant ability [67].

##### Quercetin and Rutin (Quercetin-3-*O*-Rutinoside)

They are found in many fruits and vegetables, mostly capers and radishes, and from citrus fruit, respectively. Studies on animal models of NAFLD [68,69] have largely demonstrated the efficacy of these molecules in reducing triglyceride content and oxidative injuries in fat-enriched hepatocytes. Both quercetin and rutin are able to reverse the metabolic changes induced by high fat/high-carbohydrate diet. Notably their copper-binding property have been known for a long time [60,70].

##### Resveratrol (3,5,4′-Trihydroxy-*Trans*-Stilbene)

This is known to have anti-inflammatory and antioxidative properties. Furthermore, resveratrol is also described as having therapeutic potential for preventing or treating NAFLD and insulin- resistance-related metabolic disorders. Several studies on animals demonstrate that resveratrol is useful in the prevention of liver steatosis and in its treatment. Resveratrol showed an anti-lipogenic effect by decreasing de novo lipogenesis and triacylglycerol synthesis in addition to increasing FA β-oxidation, with an overall reduction of oxidative stress [71]. Moreover the copper-binding properties of resveratrol are described [57].

**Figure 2:**
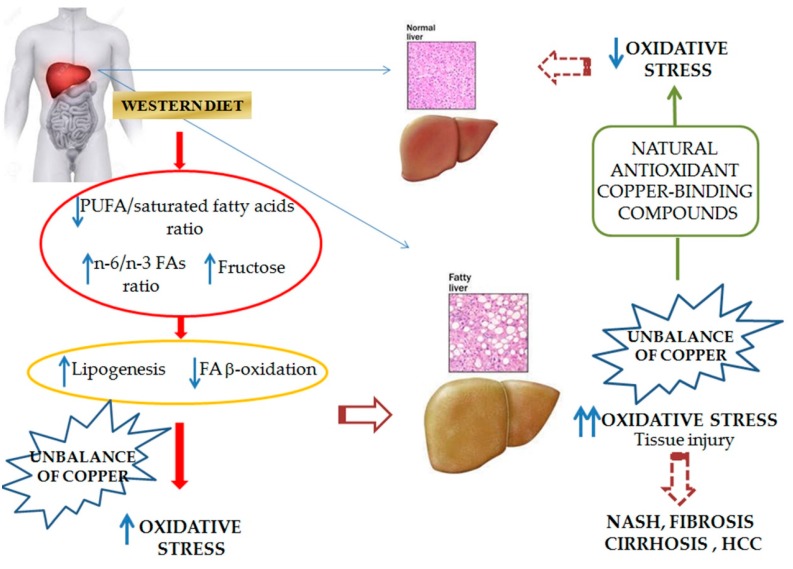
Summary of the main information on the onset and progression of NAFLD linked to diet. This highlights the role of FAs and their saturation rate, as well as fructose, which is typically found in the Western diet in increasing lipogenesis and reducing FA β-oxidation, which causes oxidative stress and eventually NAFLD. These conditions are characterized by an imbalance of copper. Some natural antioxidant compounds, which bind copper, are able to counteract NAFLD. PUFA: poly-unsaturated fatty acids.

Table 1 summarizes this information and Figure 2 resumes the concepts highlighted throughout this review.

## 4. Conclusions

NAFLD, with its progression towards NASH and more severe liver diseases, has been recognized as a pathology related to oxidative stress, with a copper imbalance having a role in its pathogenesis. Understanding the mechanism that controls the copper homeostasis is of primary importance. The mechanism of dietary copper acquisition, the molecular bases of copper delivery to tissues and the control of copper levels within the cells are topics that require detailed dissection in the near future. Of note, the Western diet is associated with high intake of fats and carbohydrates. Furthermore, it is poor in polyphenols, with devastating health consequences, such as promoting NAFLD. Many antioxidant compounds recognized as being effective against NAFLD and its progression have been demonstrated to bind copper, signaling the importance of the fine regulation of this bio-metal.

Since there is a gap between the knowledge of the chemical properties of these compounds and their therapeutic applications, this review also paves the way to broaden the research on natural antioxidant compounds against NAFLD, which considers their ability to bind copper.

## Figures and Tables

**Table 1:** Natural antioxidants described to counteract non-alcoholic fatty liver disease (NAFLD) and with copper-binding ability.

Natural Antioxidant	Food Source	Ability to Counteract NAFLD and Its Progression	Copper Binding Ability
Curcumin	Rhizomes of Curcuma longa	Salomone et al., 2016 [51] Shapiro and Bruck, 2005 [52] Inzaugarat et al., 2017 [53]	Zhao et al., 2016 [54] Zhang et al., 2010 [55]
Epigallocatechin-3-Gallate (EGCG)	Green tea	Aline B. Santamarina et al., 2015 [56]	Wing et al., 2015 [57]
Luteolin and Luteolin-7-Glucoside	Aromatic plants	Yin et al., 2017 [58] Sá et al., 2015 [59]	Brown et al., 1998 [60]
Caffeic Acid and Caffeine	Coffee	Yesil et al., 2013 [61] Shen et al., 2014 [62] Liao et al., 2014 [63]	Nkhili et al., 2014 [64]
Oleuropein	Olive and olive leaves	Barbaro et al., 2014 [65] Kim et al., 2014 [66]	Bendini et al., 2006 [67]
Quercetin and Rutin	Vegetables, mostly capers and radish, citrus fruit	Porras et al., 2016 [68] Panchal et al., 2011 [69]	Brown et al., 1998 [60] Bukhari et al., 2009 [70]
Resveratrol	Grapes	Aguirre Let al., 2014 [71]	Wing et al., 2015 [57]
